# Association of Traumatic Brain Injury With Mortality Among Military Veterans Serving After September 11, 2001

**DOI:** 10.1001/jamanetworkopen.2021.48150

**Published:** 2022-02-11

**Authors:** Jeffrey T. Howard, Ian J. Stewart, Megan Amuan, Jud C. Janak, Mary Jo Pugh

**Affiliations:** 1 University of Texas at San Antonio, San Antonio; 2 Uniformed Services University of the Health Sciences, Bethesda, Maryland; 3 Department of Internal Medicine, University of Utah School of Medicine, Salt Lake City; 4 Division of Epidemiology, US Department of Veterans Affairs, Salt Lake City, Utah; 5 Bexar Data, LLC, San Antonio, Texas

## Abstract

**Importance:**

Emerging evidence suggests that harmful exposures during military service, such as traumatic brain injury (TBI), may contribute to mental health, chronic disease, and mortality risks.

**Objective:**

To assess the mortality rates and estimate the number of all-cause and cause-specific excess deaths among veterans serving after the September 11, 2001, terrorist attacks (9/11) with and without exposure to TBI.

**Design, Setting, and Participants:**

This cohort study analyzed administrative and mortality data from January 1, 2002, through December 31, 2018, for a cohort of US military veterans who served during the Global War on Terrorism after the 9/11 terrorist attacks. Veterans who served active duty after 9/11 with 3 or more years of care in the Military Health System or had 3 or more years of care in the Military Health System and 2 or more years of care in the Veterans Health Administration were included for analysis. The study used data from the Veterans Affairs/Department of Defense Identity Repository database, matching health records data from the Military Health Service Management Analysis and Reporting tool, the Veterans Health Administration Veterans Informatics and Computing Infrastructure, and the National Death Index. For comparison with the total US population, the study used the Centers for Disease Control and Prevention WONDER database. Data analysis was performed from June 16 to September 8, 2021.

**Exposure:**

Traumatic brain injury.

**Main Outcomes and Measures:**

Multivariable, negative binomial regression models were used to estimate adjusted all-cause and cause-specific mortality rates for the post-9/11 military veteran cohort, stratified by TBI severity level, and the total US population. Differences in mortality rates between post-9/11 military veterans and the total US population were used to estimate excess deaths from each cause of death.

**Results:**

Among 2 516 189 post-9/11 military veterans (2 167 736 [86.2%] male; and 45 324 [1.8%] American Indian/Alaska Native, 160 178 [6.4%], Asian/Pacific Islander, 259 737 [10.3%] Hispanic, 387 926 [15.4%] non-Hispanic Black, 1 619 834 [64.4%] non-Hispanic White, and 43 190 [1.7%] unknown), 17.5% had mild TBI and 3.0% had moderate to severe TBI; there were 30 564 deaths. Adjusted, age-specific mortality rates were higher for post-9/11 military veterans than for the total US population and increased with TBI severity. There were an estimated 3858 (95% CI, 1225-6490) excess deaths among all post-9/11 military veterans. Of these, an estimated 275 (95% CI, −1435 to 1985) were not exposed to TBI, 2285 (95% CI, 1637 to 2933) had mild TBI, and 1298 (95% CI, 1023 to 1572) had moderate to severe TBI. Estimated excess deaths were predominantly from suicides (4218; 95% CI, 3621 to 4816) and accidents (2631; 95% CI, 1929 to 3333). Veterans with moderate to severe TBI accounted for 33.6% of total excess deaths, 11-fold higher than would otherwise be expected.

**Conclusions and Relevance:**

This military veteran cohort experienced more excess mortality compared with the total US population than all combat deaths from 9/11/01 through 9/11/21, concentrated among individuals exposed to TBI. These results suggest that a focus on what puts veterans at risk for accelerated aging and increased mortality is warranted.

## Key Points

**Question:**

Is exposure to traumatic brain injury associated with excess mortality after service in US veterans after the September 11, 2001, terrorist attacks (9/11), and what are the mortality rates among post-9/11 veterans compared with the total US population?

**Findings:**

In this cohort study of data on 2 516 189 military veterans, post-9/11 military veterans experienced excess all-cause and cause-specific mortality compared with the total US population. The numbers of excess deaths were greater among those exposed to traumatic brain injury.

**Meaning:**

These results suggest that post-9/11 military veterans have higher mortality, especially among veterans exposed to traumatic brain injury compared with the general US population and that a focus on what puts veterans at risk for increased mortality is warranted.

## Teaser

This cohort study examines the association of traumatic brain injury with mortality among US military veterans serving after the September 11, 2001, terrorist attacks and compares mortality rates for this group with rates for the total US population.

## Introduction

On August 15, 2021, in Afghanistan, Taliban forces seized the country’s capital, Kabul. Fifteen days later, on August 30, 2021, the last US military forces left Afghanistan, marking the end of almost 20 years of conflict that began with the initial response to the terrorist attacks on September 11, 2001 (9/11), and the subsequent Global War on Terrorism, which also involved conflicts in Iraq and other regional military operations. In the 20 years since 9/11, approximately 4.6 million individuals^1^ have served in the US military. As of September 3,2021, 53 283 had been wounded in combat, and 5461^2^ had died in combat while supporting the Global War on Terrorism, including 13 service members killed in Kabul on August 26, 2021, during the final evacuation. In addition, the number of post-9/11 military veterans with service-connected disabilities is estimated to be 1.8 million, more than double the number from the first Gulf War and triple the number from previous wars.^1^

While research from previous wars suggested that military veterans benefited from lower all-cause mortality than the total US population,^3,4^ more recent research suggests that this so-called healthy soldier effect may be disappearing among post-9/11 military veterans.^5^ The number and length of deployments^6^ over 20 years of war, and the subsequent psychological toll, including high prevalences of posttraumatic stress disorder (PTSD) and depression, could be contributing factors. Another factor could be the scale and impact of exposure to traumatic brain injury (TBI). Traumatic brain injury has been described as one of the signature wounds^7^ of the Global War on Terrorism, with recent estimates of the number of service members exposed to TBI numbering in excess of 430 000 since 2000.^8^ There is emerging evidence that TBI is associated with significant increases in the risk of mental health diagnoses, including PTSD,^9,10^ depression,^10^ anxiety,^10^ and dementia,^11^ as well as cardiovascular disease (CVD)^12^ and premature death.^13^ However, the extent of the all-cause and cause-specific mortality associated with exposure to TBI among post-9/11 military veterans remains unclear. We sought to assess the total all-cause and cause-specific mortality burden and estimate the total number of excess deaths among post-9/11 military veterans with and without exposure to TBI compared with the total US population.

## Methods

### Design and Study Setting

**Figure 1.**
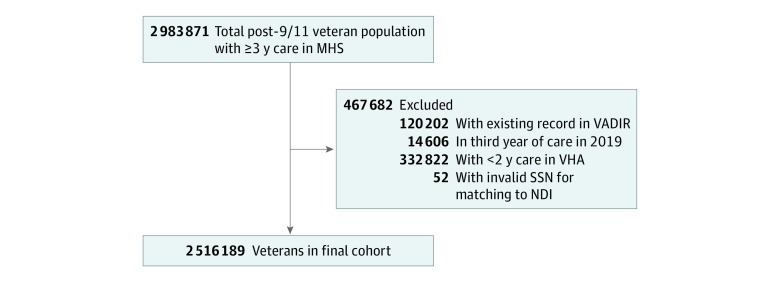
Study Flow Diagram MHS indicates Military Health System; NDI, National Death Index; SSN, Social Security number; VADIR, Veterans Affairs/Department of Defense Identity Repository; and VHA, Veterans Health Administration.

This is a cohort study of all-cause and cause-specific mortality rates for military veterans who served active duty in the US military after 9/11 and received care (outpatient, inpatient, or prescription) in the Department of Defense Military Health System (MHS) with or without care in the Veterans Health Administration (VHA) and the total US population. Both the military veteran population and the total US population were observed from January 1, 2002, to December 31, 2018. The analysis was performed from June 16 to September 8, 2021. Since military operations after 9/11 began in October 2001, no military veterans met the criteria for the study until 2002. This study was conducted as part of the Long-Term Impact of Military-Relevant Brain Injury Consortium, which aims to longitudinally examine health trajectories of veterans with and without TBI who are receiving care in the MHS only or within both the MHS and VHA. To meet this aim, veterans were required to have 3 or more years of MHS care or 3 or more years of care in the MHS plus 2 or more years of VHA care (for those who entered the VHA health care system). Individuals were eligible for inclusion upon their third year of care if they were alive and 18 years of age or older (Figure 1). Individuals without a valid social security number on file were unable to be matched to the National Death Index (NDI) and were excluded. The research protocol was approved by the University of Utah Institutional Review Board, which waived informed consent because the data were deidentified and aggregated from individual-level data for this particular study. This study followed the Strengthening the Reporting of Observational Studies in Epidemiology (STROBE) reporting guideline.

### Data Sources

Demographic and deployment data for veterans were retrieved from the Veterans Affairs/Department of Defense Identity Repository database, along with matching health records data from the MHS Management Analysis and Reporting tool and the VHA Veterans Informatics and Computing Infrastructure, and combined with data from the NDI for mortality follow-up through December 31, 2018. Population and death counts for the total US population were compiled from January 1, 2002, to December 31, 2018, from the Centers for Disease Control and Prevention WONDER (Wide-ranging Online Data for Epidemiologic Research) database. Population and death counts were aggregated by each value of year, age group, sex, race and ethnicity, deployment status, and TBI severity.

### Measures

Demographic variables for age, sex, and race and ethnicity were included in this study. Age was measured as 10-year age groups (18-24, 25-34, 35-44, 45-54, 55-64, 65-74, and 75-84 years of age). Sex was recorded as male or female. Race and ethnicity were recorded according to the US Census Bureau bridged race and ethnicity categories (American Indian/Alaska Native, Asian/Pacific Islander, Hispanic, non-Hispanic Black, and non-Hispanic White, and unknown). Each year of observation was coded as numeric year, 2002 to 2018, with each year beginning on January 1 and endeding on December 31.

For veterans, 2 additional variables were included to account for deployment status and exposure to and severity of TBI, and since deployment was not applicable and TBI exposure was not known, the total US population was coded as a separate category (nonveteran). Deployment status was measured as (1) total US population, (2) veteran not deployed, and (3) veteran deployed. Deployment status was determined based on the presence of a deployment indicator in the Veterans Affairs/Department of Defense Identity Repository database. Traumatic brain injury was measured as one of 4 categories: (1) total US population, (2) veteran no TBI, (3) veteran with mild TBI, (4) veteran with moderate to severe TBI, consistent with prior research.^10^ Military veterans were categorized as no TBI if there was no known record of either a positive result on the TBI screening from the Clinical Reminder and Comprehensive TBI Evaluation (CTBIE) protocol, or a medical record diagnosis of mild, moderate, severe, or penetrating TBI (eTable 1 in the Supplement). For military veterans with a positive result on the CTBIE screening, severity was determined based on self-reported loss of consciousness (LOC), alteration of consciousness (AOC), or posttraumatic amnesia (PTA) using the following definitions: mild (LOC <30 minutes; AOC = 24 hours; PTA = 24 hours); moderate (LOC >30 minutes and <24 hours; AOC >24 hours; PTA >24 hours and <7 days); severe (LOC >24 hours; AOC >24 hours; PTA >7 days). For military veterans without a positive result on the CTBIE screening, we used the *International Classification of Diseases, Ninth Revision (ICD-9)* and *International Statistical Classification of Diseases and Related Health Problems, Tenth Revision (ICD-10)* coding to identify TBI exposure and severity based on the Armed Forces Health Surveillance Branch definitions^10^ (eTable 2 in the Supplement). Veterans with positive screening for TBI but no medical record of TBI severity, or who had a medical record diagnosis of mild TBI or TBI of unknown or unclassified severity were categorized as mild TBI. Veterans who had a medical diagnosis of moderate, severe, or penetrating TBI were categorized as moderate to severe TBI.

Causes of death were determined from underlying cause of death *ICD-9* and *ICD-10* codes supplied by the NDI (eTable 3 in the Supplement). The top 5 most frequent cause of death categories were then coded into separate specific causes for analysis: accident, suicide, cancer, CVD, and homicide. All other causes of death were grouped as all other causes.

### Statistical Analysis

Descriptive statistics are reported as total population and death counts, along with category percentages and crude mortality rates per 100 000 person-years. Adjusted mortality rates per 100 000 person-years were estimated using multivariable negative binomial regression models with population counts as denominators for veterans and the total US population. Covariates used to control confounding included age groups, sex, race and ethnicity, indicators of deployment status, and severity of TBI. Mortality rates and 95% CIs are reported graphically. Excess deaths were calculated as the difference in mortality rates between the post-9/11 military veteran population overall and the TBI severity subgroups and the total US population, multiplied by each group’s population per 100 000 person-years. Excess deaths were reported as the estimated number of excess deaths with 95% CI. A 2-tailed *P* < .05 was considered statistically significant. Data were analyzed using R, version 4.0.2 (R Foundation for Statistical Computing).

## Results

A total of 2 516 189 individual military veterans (2 167 736 [86.2%] male; 45 324 [1.8%] American Indian/Alaska Native, 160 178 [6.4%] Asian/Pacific Islander, 259 737 [10.3%] Hispanic, 387 926 [15.4%] non-Hispanic Black, 1 619 834 [64.4%] non-Hispanic White, and 43 190 [1.7%] unknown [which included anyone not listed in the other 5 groups]) were included in the analysis, accounting for 16 071 373 person-years of observation, and 30 564 deaths between 2002 and 2018 (Table 1; eTable 4 in the Supplement). Among the military veterans, 1 999 729 (79.5%) had no known exposure to TBI, 441 083 (17.5%) were exposed to mild TBI, and 75 377 (3.0%) were exposed to moderate to severe TBI. A total of 3 894 781 528 person-years were observed for the total US population from 2002 through 2018, with 29 445 853 total deaths. The military veteran population was composed primarily of those ages 18-44, men, and non-Hispanic White veterans. Approximately three-fourths of the military veteran population (1 869 256 [74.6%]) were deployed sometime after 9/11. Individuals with TBI exposure, both mild (384 624 [87.2%] of 441 083) and moderate to severe (64 824 [86.0%] of 75 377), were more likely to have been deployed than individuals with no TBI exposure.

**Table 1.** Descriptive Statistics of Post-9/11 Military Veteran Population by Traumatic Brain Injury Severity and Total US Population, 2002 to 2018

Variables	No. (%)				
	Veteran population				Total US population
	Total	No TBI	Mild TBI	Moderate to severe TBI	
No.	2 516 189	1 999 729 (79.5)	441 083 (17.5)	75 377 (3.0)	229 104 796^a^
Person-years	16 071 373	12 460 025	3 100 813	510 535	3 894 781 528
Age, y					
18-24	601 841 (23.9)	481 061 (24.1)	103 245 (23.4)	17 535 (23.3)	30 241 833 (13.2)
25-34	1 159 053 (46.1)	899 860 (45.0)	221 823 (50.3)	37 370 (49.6)	41 697 073 (18.2)
35-44	440 253 (17.5)	350 730 (17.5)	76 138 (17.3)	13 385 (17.8)	41 926 178 (18.3)
45-54	234 028 (9.3)	197 119 (9.9)	31 518 (7.1)	5391 (7.2)	43 071 702 (18.8)
55-64	68 175 (2.7)	59 642 (3.0)	7159 (1.6)	1374 (1.8)	35 740 348 (15.6)
65-74	12 782 (0.5)	11 267 (0.6)	1194 (0.3)	321 (0.4)	22 910 480 (10.0)
75-84	58 (0)	50 (0)	6 (0)	2 (0)	13 517 183 (5.9)
Sex					
Male	2 167 736 (86.2)	1 696 396 (84.8)	401 894 (91.1)	69 446 (92.1)	112 261 350 (49.0)
Female	348 453 (13.8)	303 333 (15.2)	39 189 (8.9)	5931 (7.9)	116 843 446 (51.0)
Race and ethnicity					
American Indian/Alaska Native	45 324 (1.8)	34 898 (1.7)	8926 (2.0)	1500 (2.0)	1 832 838 (0.8)
Asian/Pacific Islander	160 178 (6.4)	119 702 (6.0)	34 209 (7.8)	6267 (8.3)	12 371 659 (5.4)
Hispanic	259 737 (10.3)	205 892 (10.3)	46 496 (10.5)	7349 (9.7)	32 761 986 (14.3)
Non-Hispanic Black	387 926 (15.4)	315 533 (15.8)	62 803 (14.2)	9590 (12.7)	27 950 785 (12.2)
Non-Hispanic White	1 619 834 (64.4)	1 284 606 (64.2)	285 100 (64.6)	50 128 (66.5)	154 187 528 (67.3)
Unknown^b^	43 190 (1.7)	39 098 (2.0)	3549 (0.8)	543 (0.7)	0
Deployment status					
Deployed	1 869 256 (74.6)	1 419 808 (71.0)	384 624 (87.2)	64 824 (86.0)	NA
Not deployed	646 933 (25.4)	579 921 (29.0)	56 459 (12.8)	10 553 (14.0)	NA
Total deaths, sum	30 564	21 561	6989	2014	29 445 853
Crude mortality rates per 100 000 person-years					
All causes^c^	190.2	173.0	225.4	394.5	756.0
Accident	53.7	42.6	81.5	154.3	46.0
Suicide	38.2	34.3	46.9	81.5	15.9
Cancer	32.6	34.8	23.0	35.6	210.0
CVD	22.7	22.5	22.1	31.5	221.1
Homicide	10.8	10.4	10.4	23.9	6.9
All other causes	32.2	28.4	41.5	67.6	256.1

Abbreviations: CVD, cardiovascular disease; NA, not applicable; TBI, traumatic brain injury.

^a^ Mean number of unique individuals in total population per year.

^b^ Included anyone not listed in the other 5 groups.

^c^ Totals for each column may not sum to 100 because of rounding.

Crude all-cause mortality rates for military veterans were 173.0 per 100 000 person-years for the no TBI group, 225.4 per 100 000 person-years for the mild TBI group, and 394.5 per 100 000 person-years for the moderate to severe TBI group, which were lower than the crude all-cause mortality rate for the total US population (756.0) (Table 1). The top 5 causes of death for military veterans were accident (53.7 per 100 000 person-years), suicide (38.2 per 100 000 person-years), cancer (32.6 per 100 000 person-years), CVD (22.7 per 100 000 person-years), and homicide (10.8 per 100 000 person-years). Mortality rates for each cause of death were higher among veterans with moderate to severe TBI compared with veterans with no TBI (accident: 154.3 per 100 000 person-years vs 42.6 per 100 000 person-years; suicide: 81.5 per 100 000 person-years vs 34.3 per 100 000 person-years; cancer: 35.6 per 100 000 person-years vs 34.8 per 100 000 person-years; CVD: 31.5 per 100 000 person-years vs 22.5 per 100 000 person-years; homicide: 23.9 per 100 000 person-years vs 10.4 per 100 000 person-years). The total US population had lower crude mortality rates for accident (46.0 per 100 000 person-years), suicide (15.9 per 100 000 person-years), and homicide (6.9 per 100 000 person-years) but higher crude mortality rates for cancer (210.0 per 100 000 person-years) and CVD (221.1 per 100 000 person-years) compared with military veterans, regardless of TBI exposure.

**Figure 2.**
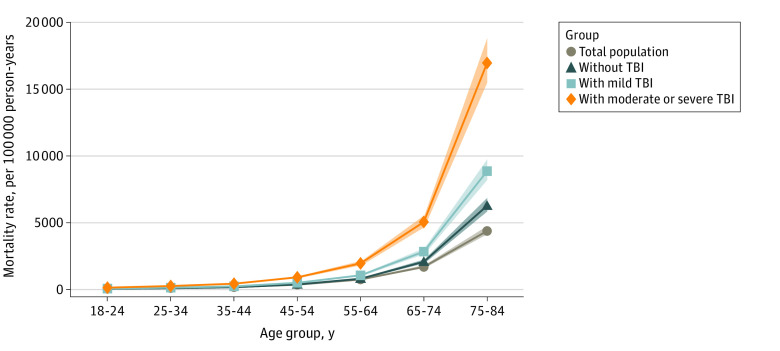
Multivariable Adjusted, Age-Specific All-Cause Mortality Rates per 100 000 Person-Years by Population Subgroups Shaded areas indicate 95% CIs.

**Figure 3.**
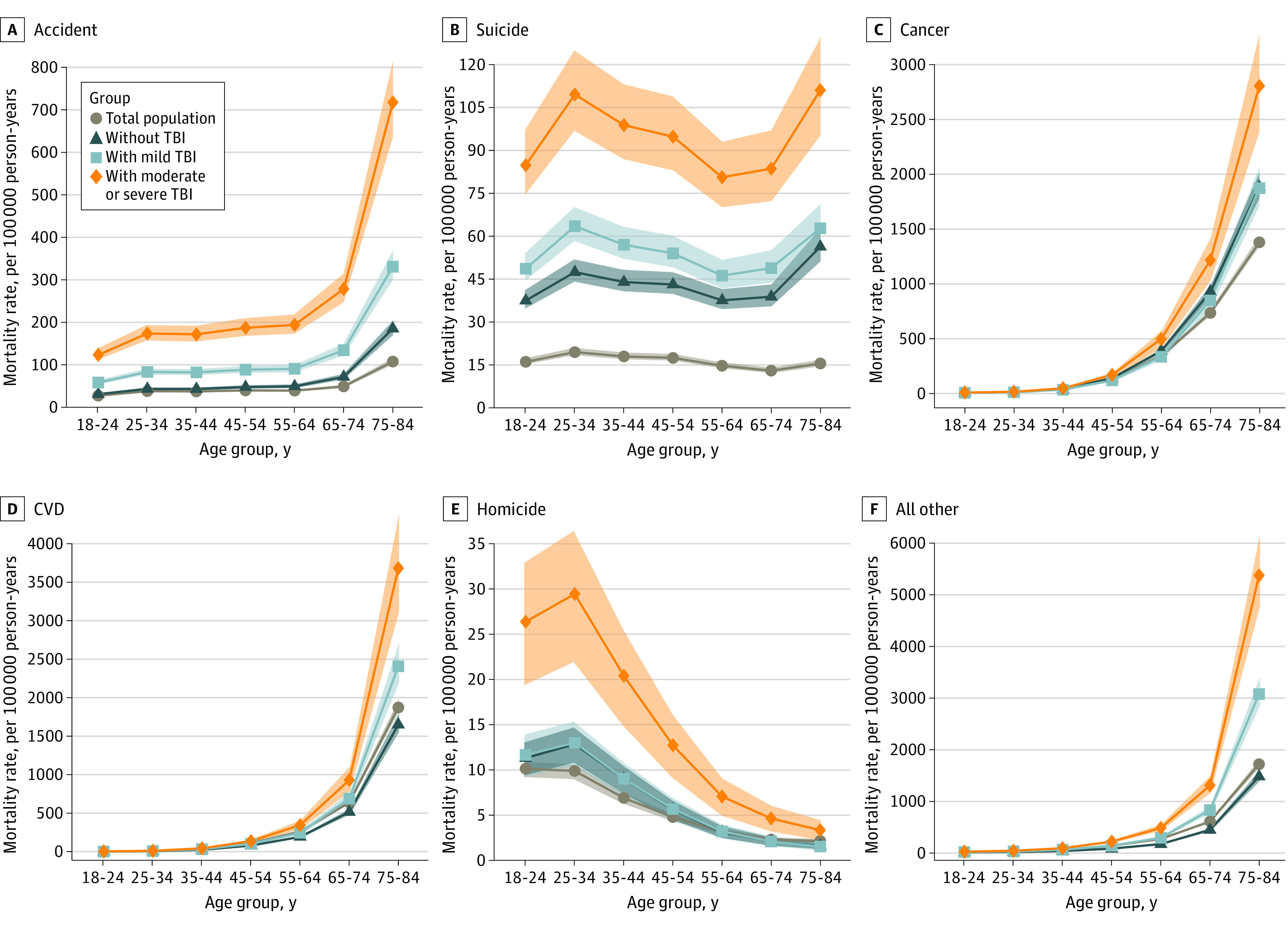
Multivariable Adjusted, Age-Specific Mortality Rates per 100 000 Person-Years Shaded areas indicate 95% CIs; CVD, cardiovascular disease; TBI, traumatic brain injury.

Multivariable adjusted, age-specific, all-cause mortality rates were higher for military veterans than for the total US population and increased monotonically with increasing TBI severity (Figure 2). All-cause mortality rate differences between veterans and the total US population were greatest among older veterans (among veterans 75-84 years with moderate to severe TBI: 17 009.5 per 100 000 person-years [95% CI: 15 367.4-18 651.5) vs veterans with mild TBI: 8902.0 per 100 000 person-years [95% CI: 8138.8 to 9665.3] and veterans without TBI: 6305.7 per 100 000 person-years [95% CI: 5804.0 to 6807.5] vs total population: 4414.6 per 100 000 person-years [95% CI: 4112.2 to 4716.9]). Similar patterns were observed for each of the top 5 causes of death, with adjusted mortality rates significantly higher among military veterans exposed to mild and moderate to severe TBI consistently showing higher mortality rates than veterans with no TBI and the total US population (Figure 3). Differences in mortality rates between veterans with and without TBI and the total US population tended to be greatest in older ages, with the exception of homicide, which was greatest in the group aged 25 to 34 years (veterans with moderate to severe TBI: 29.5 per 100 000 person-years [95% CI: 22.2-36.8] vs veterans with mild TBI: 13.0 per 100 000 person-years [95% CI: 10.5-15.5] vs veterans without TBI: 12.9 per 100 000 person-years [95% CI: 10.9-14.8] vs total population: 9.9 per 100 000 person-years [95% CI: 9.1-10.7]. Adjusted mortality rates for accident, suicide, and homicide were especially elevated among military veterans with exposure to moderate to severe TBI.

There were an estimated 3858 (95% CI, 1225 to 6490) excess deaths among all veterans (Table 2). Of these, 275 (95% CI, −1435 to 1985) were not exposed to TBI, 2285 (95% CI, 1637 to 2933) had mild TBI, and 1298 (95% CI, 1023 to 1572) had moderate to severe TBI. Most excess deaths were concentrated in individuals 18 to 44 years of age. In fact, there were an estimated 4328 (95% CI, 2482 to 6174) excess deaths in total among veterans aged 18 to 44 years. Similarly, there were an estimated 2631 (95% CI, 1929 to 3333) excess deaths from accident, 4218 (95% CI, 3621 to 4816) from suicide, 378 (95% CI, 27 to 729) from cancer, and 415 (95% CI, 99 to 732) from homicide. Most excess deaths for these causes were among veterans 18 to 44 years of age, with the exception of cancer, which was concentrated among veterans 45 to 84 years of age.

**Table 2.** Total and Cause-Specific Excess Death Estimates for Post-9/11 Military Veteran Population by Age Group and Traumatic Brain Injury Severity, 2002 to 2018

Cause of death by age group	Veteran population (95% CI)			
	Total	No TBI	Mild TBI	Moderate to severe TBI
**Accident**				
18-44 y	2266 (1666 to 2865)	520 (179 to 861)	1168 (987 to 1349)	578 (501 to 655)
45-84 y	365 (263 to 468)	151 (82 to 220)	141 (118 to 164)	73 (63 to 83)
Total	2631 (1929 to 3333)	671 (261 to 1082)	1309 (1105 to 1513)	651 (564 to 738)
**Suicide**				
18-44 y	3736 (3210 to 4263)	2418 (2079 to 2757)	985 (851 to 1120)	333 (281 to 386)
45-84 y	482 (411 to 554)	364 (311 to 417)	87 (74 to 100)	31 (26 to 36)
Total	4218 (3621 to 4816)	2782 (2398 to 3175)	1072 (924 to 1220)	364 (307 to 422)
**Cancer**				
18-44 y	2 (−116 to 120)	26 (−55 to 107)	−38 (−64 to −12)	14 (3 to 24)
45-84 y	376 (143 to 609)	356 (177 to 535)	−13 (−50 to 23)	33 (16 to 50)
Total	378 (27 to 729)	382 (122 to 642)	−51 (−114 to 12)	47 (19 to 75)
**CVD**				
18-44 y	−564 (−674 to −455)	−512 (−580 to −443)	−60 (−90 to −31)	8 (−4 to 19)
45-84 y	−638 (−813 to −463)	−638 (−764 to −512)	−21 (−55 to 13)	21 (6 to 36)
Total	−1202 (−1487 to −918)	−1150 (−1344 to −955)	−81 (−145 to −18)	29 (2 to 55)
**Homicide**				
18-44 y	400 (104 to 696)	248 (50 to 447)	71 (5 to 137)	81 (50 to 113)
45-84 y	15 (−5 to 35)	10 (−6 to 25)	2 (−1 to 5)	3 (2 to 5)
Total	415 (99 to 732)	258 (44 to 472)	73 (4 to 142)	84 (51 to 117)
**All other causes**				
18-44 y	−1512 (−1709 to −1315)	−1501 (−1615 to −1386)	−69 (−128 to −9)	58 (34 to 81)
45-84 y	−1071 (−1254 to −887)	−1168 (−1292 to −1044)	33 (−8 to 74)	65 (46 to 83)
Total	−2583 (−2963 to −2202)	−2669 (−2907 to −2430)	−36 (−137 to 64)	122 (80 to 164)
**Total excess deaths**				
18-44 y	4328 (2482 to 6174)	1200 (57 to 2343)	2057 (1561 to 2554)	1071 (864 to 1277)
45-84 y	−470 (−1257 to 316)	−925 (−1492 to −357)	228 (77 to 379)	227 (159 to 294)
Total	3858 (1225 to 6490)	275 (−1435 to 1985)	2285 (1637 to 2933)	1298 (1023 to 1572)

Abbreviations: CVD, cardiovascular disease; TBI, traumatic brain injury.

For veterans not exposed to TBI, large mortality advantages were observed for CVD (−1150; 95% CI, −1344 to −955) and other causes (−2669, 95% CI, −2907 to −2430). Veterans exposed to mild TBI had a mortality advantage for CVD (−81, 95% CI, −145 to −18); differences found for cancer (−51, 95% CI, −114 to 12) and other causes (−36, 95% CI, −137 to 64) were not significant. In contrast, veterans exposed to moderate to severe TBI had excess deaths across all causes of death and accounted for a disproportionate number of total excess deaths: 1298 (95% CI, 1023 to 1572) of 3858 (1225 to 6490). While veterans exposed to moderate to severe TBI accounted for only 3.0% of the total post-9/11 military veteran population, they accounted for 33.6% of total excess deaths observed, 11-fold higher than would otherwise be expected.

## Discussion

To our knowledge, this cohort study is the first study to estimate the number of excess deaths among a large cohort of over 2.5 million post-9/11 military veterans receiving care in the MHS and VHA. The findings suggest that our cohort has higher adjusted mortality rates for death from all causes, accident, suicide, and homicide compared with the total US population. Veterans exposed to TBI had higher mortality rates than veterans who were unexposed as well as the total US population. Exposure to moderate to severe TBI was associated with the highest mortality rates and excess deaths across all causes of death.

Prior research on veterans of World War II^3^ and the Persian Gulf War^4,14^ found lower mortality among military veterans who served in those wars. A more recent study among active duty and military veterans who served between 2002 and 2007 found that only suicide rates were elevated above those in the US total population.^15^ In contrast, our findings are consistent with another more recent study that found post-9/11 military veterans had higher all-cause mortality than the total US population^5^ and that excess mortality was concentrated among younger veterans, aged 18 to 44 years. In our cohort, excess deaths were observed from accident, suicide, cancer, and homicide, although suicide and accidental deaths were by far the biggest contributors. In fact, these 2 causes of death were associated with an estimated 6849 excess deaths, 1393 more deaths than the total number of combat deaths experienced over 20 years of war.

Our findings suggest important patterns highlighting increased health risks for veterans. While our data are not detailed enough to fully explore the reasons for increased risk, some possible reasons can be inferred from prior research. For example, high rates of suicide have been previously reported for post-9/11 military veterans.^16^ Suicide among military veterans is associated with risk factors that are also common in the civilian population, including PTSD^17,18^ and depression,^17,18^ as well as with financial and relationship problems.^19^ Military-specific risk factors associated with suicide may include a lack of social support after separation from the military,^18,20^ anger,^17,20^ exposure to combat,^21,22^ witnessing the killing of others,^21,22^ and other traumatic exposures.^22,23^ In addition, high rates of accidental injury and death^15,24^ have been reported among military veterans, particularly among younger veterans and after separation from the military. Our results were also consistent with this pattern. Excess mortality among young military veterans may be associated with a tendency toward engaging in risky behavior,^14,25,26^ including drug and alcohol use, speeding, driving under the influence, and not wearing seat belts. Similarly, the higher mortality rates and excess deaths from homicide among post-9/11 military veterans in this study may also be associated with issues of difficulty in transitioning to civilian life after military service because transition difficulty is associated with risk-taking behavior, PTSD, anger, and impulse control.^25,27^ In particular, increased risk of homicide for military veterans is strongly associated with substance use disorders, including both drugs and alcohol,^28^ which have high prevalence in the military veteran population.^29^ Environmental exposures, such as burn pits and other toxic chemicals,^30,31^ may help explain the higher cancer mortality rates observed in the post-9/11 military veteran population.

Our results also suggest that exposure to TBI, especially moderate to severe TBI, exacerbates mortality risks seen in our total cohort. Veterans in our cohort who were exposed to moderate to severe TBI had the highest mortality rates across all causes of death, including CVD and other causes, and disproportionately high numbers of estimated excess deaths. This is consistent with other findings showing that combat injury is associated with long-term health risks for military veterans, including hypertension, coronary artery disease, diabetes, and chronic kidney disease.^32,33,34^ Exposure to TBI, specifically, has also been associated with increased risk of mental health issues,^10^ including PTSD, anxiety, and depression, which in turn are associated with suicide and accident-related mortality.^35^ Emerging research has also begun to suggest associations between TBI and other chronic diseases, including CVD.^36^ Our findings provide further support for associations between moderate to severe TBI and increased cardiovascular mortality and other chronic disease mortality. There are several possible mechanisms in these associations, including accelerated cellular aging,^37,38^ chronic inflammation,^39^ behavioral factors,^40,41^ and neurological and cognitive decline.^42^

Compared with the total US population, veterans in our cohort with no known exposure to TBI also experienced excess mortality from suicide, accidents, cancer, and homicide. Conversely, this group had large mortality advantages for CVD and other causes of death, which likely reflect selection processes generally associated with better health.^3,43^ These mortality advantages were enough to offset excess deaths from suicide, accidents, cancer, and homicide overall and among older veterans aged 45 to 84 years, but not among younger veterans aged 18 to 44 years. In fact, the already measurable excess mortality in this relatively young cohort may be a warning of an even larger burden of chronic disease and premature mortality in the future as this cohort ages. Although mortality data on TBI exposure are not available for the total US population, it is reasonable to expect similar increases in mortality rates for civilians with TBI exposures. Thus, these results have implications that extend beyond the military veteran population.

### Strengths and Limitations

This study has several strengths, including the use of longitudinal merged health system data from the VA and MHS on a large cohort of post-9/11 military veterans, including those who did not seek VA care, and the use of mortality data from the NDI for establishing underlying causes of death.

The study also has limitations. First, our inclusion criteria required 3 years of MHS data, which excluded individuals who left military service without care in 3 years. Second, while the use of death certificate–based cause of death coding is common, there is potential for cause of death misclassification. Third, TBI diagnoses and severity levels are approximations and may not fully represent the exposure to or severity of injuries sustained. In addition, methods for identifying TBI were enhanced with the CTBIE; however, limitations persist, particularly for mild TBI. Fourth, this study compared post-9/11 military veteran cohort with the total US population, which may also lead to underestimates of excess mortality associated with healthy selection bias among the military cohort.

## Conclusions

Despite historically low combat fatality rates observed in Iraq and Afghanistan,^44^ our study suggests that post-9/11 military veterans face a higher mortality burden across multiple causes of death than the total US population. We also found that exposure to moderate to severe TBI was associated with even higher mortality rates and excess mortality from accident, suicide, cancer, CVD, homicide, and other causes. After 20 years of war, it is vital to focus attention on what puts veterans at risk for accelerated aging and increased mortality, as well as how it can be mitigated.

## Notes

Supplement. **eTable 1.** Frequency of Traumatic Brain Injury (TBI) Severity Level Categories and Subcategories Within the Post-9/11 Military Veteran Population **eTable 2.** List of *International Classification of Diseases, Ninth Revision (ICD-9)* and *International Statistical Classification of Diseases and Related Health Problems, Tenth Revision (ICD-10)* Codes for Traumatic Brain Injury (TBI) and Severity Levels **eTable 3.** *International Statistical Classification of Diseases and Related Health Problems, Tenth Revision (ICD-10)* Underlying Cause of Death Codes and Cause of Death Groups **eTable 4.** Number of Observations of Traumatic Brain Injury (TBI) Severity Level Categories by Year
