# Supplementary material for: Association of Traumatic Brain Injury With Mortality Among Military Veterans Serving After September 11, 2001
Source: JAMA Netw Open. 2022 Feb 11;5(2):e2148150. doi: 10.1001/jamanetworkopen.2021.48150 (PMC8837911; doi:10.1001/jamanetworkopen.2021.48150)
Supplement: Supplement. — eTable 1. Frequency of Traumatic Brain Injury (TBI) Severity Level Categories and Subcategories Within the Post-9/11 Military Veteran Population eTable 2. List of International Classification of Diseases, Ninth Revision (ICD-9) and International Statistical Classification of Diseases and Related Health Problems, Tenth Revision (ICD-10) Codes for Traumatic Brain Injury (TBI) and Severity Levels eTable 3. International Statistical Classification of Diseases and Related Health Problems, Tenth Revision (ICD-10) Underlying Cause of Death Codes and Cause of Death Groups eTable 4. Number of Observations of Traumatic Brain Injury (TBI) Severity Level Categories by Year [file jamanetwopen-e2148150-s001.pdf]

Supplemental Online Content

Howard JT, Stewart IJ, Amuan M, Janak JC, Pugh MJ. Association of traumatic brain injury with mortality among military veterans serving after September 11, 2001. *JAMA Netw Open*. 2022;5(2):e2148150. doi:10.1001/jamanetworkopen.2021.48150

eTable 1. Frequency of Traumatic Brain Injury (TBI) Severity Level Categories and Subcategories Within the Post-9/11 Military Veteran Population

eTable 2. List of *International Classification of Diseases, Ninth Revision (ICD-9)* and *International Statistical Classification of Diseases and Related Health Problems, Tenth Revision (ICD-10)* Codes for Traumatic Brain Injury (TBI) and Severity Levels

eTable 3. *International Statistical Classification of Diseases and Related Health Problems, Tenth Revision (ICD-10)* Underlying Cause of Death Codes and Cause of Death Groups

eTable 4. Number of Observations of Traumatic Brain Injury (TBI) Severity Level Categories by Year

This supplemental material has been provided by the authors to give readers additional information about their work.

© 2022 Howard JT et al. *JAMA Network Open.*

eTable 1. Frequency of traumatic brain injury (TBI) severity level categories and sub-categories within the post-9/11 military veteran population.

TBI Severity Level	TBI Detail Level	Number (%)
No TBI	No known TBI	1,999,729 (79.5)
Mild TBI	Mild<br>Initial Screen – Historical Resolved<br>Initial Screen – Positive no additional Evidence<br>Unclassified (V1552)	293,786 (11.7)<br>8,562 (0.3)<br>65,791 (2.6)<br>72,944 (2.9)
Moderate/Severe	Moderate/Severe<br>Penetrating	57,745 (2.3)<br>17,632 (0.7)
Total		2,516,189

eTable 2. List of International Classification of Diseases (ICD) version 9 and 10 codes for Traumatic Brain Injury (TBI) and severity levels.

TBI Severity Level	ICD version 9	ICD version 10
No TBI	No ICD Record	No ICD Record
Mild TBI	Mild: 80000, 80001, 80002, 80006, 80009, 80050, 80051, 80052, 80100, 80101, 80102, 80106, 80109, 80150, 80151, 80152, 80300, 80301, 80302, 80306, 80309, 80350, 80351, 80352, 80400, 80401, 80402, 80406, 80409, 80450, 80451, 80452, 8500, 8501, 85011, 3102, 95901, 850, 8505, 8509<br>Unclassified: 9501, 9502, 9503, 9070, 85400	Mild: F0781, S020XXA, S020XXB, S0210XA, S0210XB, S0291XA, S0291XB, S060X0A, S060X1A, S060X9A, S098XXA, S0990XA<br>Unclassified: S0402XA, S04039A, S04049A, S06890A, S069X9S
Moderate/ Severe	Moderate: 80003, 80010, 80011, 80012, 80013, 80016, 80019, 80020, 80021, 80022, 80023,80026, 80029, 80030, 80032, 80031, 80033, 80036, 80039, 80040, 80041, 80042, 80043, 80046, 80049, 80053, 80056,80059, 80103, 80110, 80111, 80112, 80113, 80116, 80119,80120, 80121, 80122, 80123, 80126, 80129, 80130, 80131, 80132, 80133, 80136, 80139,80140, 80141, 80142, 80143, 80146, 80149, 80153, 80156, 80159, 80303, 80310, 80311, 80312, 80313, 80316, 80319, 80320, 80321, 80322, 80323, 80326, 80329, 80330, 80331, 80332, 80333, 80336, 80339, 80340, 80341, 80342, 80343, 80346, 80349, 80353, 80356, 80359, 80403, 80410, 80411, 80412, 80413, 80416, 80419, 80420, 80421, 80422, 80423, 80426, 80429, 80430,	Moderate: S020XXA, S020XXB, S0210XA, S0210XB, S0291XA, S0291XB, S060X0A, S060X2A, S060X3A, S060X4A, S061X0A, S061X1A, S061X2A, S061X3A, S061X4A, S061X9A, S06330A, S06331A, S06332A, S06333A, S06334A, S06339A, S06360A, S06361A, S06362A, S06363A, S06364A, S06369A, S06370A, S06371A, S06372A, S06373A, S06374A, S06379A, S06380A, S06381A, S06382A, S06383A, S06384A, S06389A, S064X0A, S064X1A, S064X2A, S064X3A, S064X4A, S064X9A, S065X0A, S065X1A, S065X2A, S065X3A, S065X4A, S065X9A, S066X0A, S066X1A, S066X2A, S066X3A, S066X4A, S066X9A, S06890A, S06891A, S06892A, S06893A, S06894A, S06899A, S069X0A, S069X1A, S069X2A, S069X3A, S069X4A, S069X9A<br>Severe: S020XXA, S020XXB, S0210XA, S0210XB, S0291XA, S0291XB, S060X5A,

© 2022 Howard JT et al. *JAMA Network Open.*

	80431, 80432, 80433, 80436, 80439, 80440, 80441, 80442, 80443, 80446, 80449, 80453, 80456, 80459, 85012, 8502, 85100, 85101, 85102, 85103, 85106, 85109, 85120, 85121, 85122, 85123, 85126, 85129, 85140, 85141, 85142, 85143, 85146, 85149, 85160, 85161, 85162, 85163, 85166, 85169, 85180, 85181, 85182, 85183, 85186, 85189, 85200, 85201, 85202, 85203, 85206, 85209, 85220, 85221, 85222, 85223, 85226, 85229, 85240, 85241, 85242, 85243, 85246, 85249, 85300, 85301, 85302, 85303, 85306, 85309, 85401, 85402, 85403, 85406, 85409<br>Severe: 80004, 80005, 80014, 80015, 80024, 80025, 80034, 80035, 80044, 80045, 80054, 80055, 80104, 80105, 80114, 80115, 80124, 80125, 80134, 80135, 80144, 80145, 80154, 80155, 80304, 80305, 80314, 80315, 80324, 80325, 80334, 80335, 80344, 80345, 80354, 80355, 80404, 80405, 80414, 80415, 80424, 80425, 80434, 80435, 80444, 80445, 80454, 80455, 8503, 8504, 85104, 85105, 85124, 85125, 85144, 85145, 85164, 85165, 85184, 85185, 85204, 85205, 85224, 85225, 85244, 85245, 85304, 85305<br>85404, 85405<br>Penetrating: 8006X, 8007X, 8008X, 8009X, 8016X, 8017X, 8018X, 8019X, 8036X, 8037X, 8038X, 8039X<br>8046X, 8047X, 8048X, 8049X, 8511X, 8513X, 8515X, 8517X, 8519X, 8521X, 8523X, 8525X,<br>8531X, 8541X	S060X6A, S061X5A, S061X6A, S061X7A, S061X8A, S06335A, S06336A, S06337A, S06338A, S06365A, S06366A, S06367A, S06368A, S06375A, S06376A, S06377A, S06378A, S06385A, S06386A, S06387A, S06388A, S064X5A, S064X6A, S064X7A, S064X8A, S065X5A, S065X6A, S065X7A, S065X8A, S066X5A, S066X6A, S066X7A, S066X8A, S06895A, S06896A, S06897A, S06898A, S069X5A, S069X6A, S069X7A, S069X8A<br>Penetrating: S0190XA, S020XXB, S0210XB, S0291XB, S06330A, S06331A, S06332A, S06333A, S06334A, S06335A, S06336A, S06337A, S06338A, S06339A, S06360A, S06361A, S06362A, S06363A, S06364A, S06365A, S06366A, S06367A, S06368A, S06369A, S06370A, S06371A, S06372A, S06373A, S06374A, S06375A, S06376A, S06377A, S06378A, S06379A, S06380A, S06381A, S06382A, S06383A, S06384A, S06385A, S06386A, S06387A, S06388A, S06389A, S064X0A, S064X1A, S064X2A, S064X3A, S064X4A, S064X5A, S064X6A, S064X7A, S064X8A, S064X9A, S065X0A, S065X1A, S065X2A, S065X3A, S065X4A, S065X5A, S065X6A, S065X7A, S065X8A, S065X9A, S066X0A, S066X1A, S066X2A, S066X3A, S066X4A, S066X5A, S066X6A, S066X7A, S066X8A, S066X9A, S06890A, S06891A, S06892A, S06893A, S06894A, S06895A, S06896A, S06897A, S06898A, S06899A, S069X0A, S069X1A, S069X2A, S069X4A, S069X5A, S069X6A, S069X7A, S069X8A, S069X9A

eTable 3. International Classification of Diseases (ICD) version 10 underlying cause of death codes and cause of death groups.

Cause of Death Group	ICD-10 Underlying Cause of Death Codes
Accident	ICD 10: S00-T88, and V00-X59, Y86
Suicide	ICD 10: U03, X60-X84, Y87.0
Cancer	ICD 10: C00-D49
Cardiovascular Disease	ICD 10: I00-I99
Homicide	ICD 10: U01-U02, X85-Y09, Y87.1
All Other	All other ICD 10 codes

© 2022 Howard JT et al. *JAMA Network Open.*

eTable 4. Number of observations of traumatic brain injury (TBI) severity level categories by year.

Year	Total	No TBI	Mild TBI	Moderate/ Severe TBI
2002	588	430	126	32
2003	955	681	196	78
2004	3,172	2290	664	218
2005	13,484	9703	2,861	920
2006	361,309	272,457	75,615	13,237
2007	613,399	462,129	129,334	21,936
2008	797,959	599,906	169,764	28,289
2009	955,712	719,872	202,648	33,192
2010	1,099,288	831,234	230,616	37,438
2011	1,227,961	933,811	253,115	41,035
2012	1,341,494	1,027,512	270,100	43,882
2013	1,435,373	1,108,124	281,415	45,834
2014	1,518,126	1,181,661	289,286	47,179
2015	1,594,462	1,250,937	295,130	48,395
2016	1,654,441	1,306,715	298,547	49,179
2017	1,706,839	1,356,643	300,454	49,742
2018	1,746,811	1,395,920	300,942	49,949

© 2022 Howard JT et al. *JAMA Network Open.*
